# A cell autonomous torsinA requirement for cholinergic neuron survival and motor control

**DOI:** 10.7554/eLife.36691

**Published:** 2018-08-17

**Authors:** Samuel S Pappas, Jay Li, Tessa M LeWitt, Jeong-Ki Kim, Umrao R Monani, William T Dauer

**Affiliations:** 1 Department of NeurologyUniversity of MichiganAnn ArborUnited States; 2 Cell and Molecular Biology ProgramUniversity of MichiganAnn ArborUnited States; 3 Department of Cell BiologyColumbia University Medical CenterNew YorkUnited States; 4 Center for Motor Neuron Biology and DiseaseColumbia University Medical CenterNew YorkUnited States; 5 Department of PathologyColumbia University Medical CenterNew YorkUnited States; 6 Department of Cell and Developmental BiologyUniversity of MichiganAnn ArborUnited States; University of California, San FranciscoUnited States; Texas Children's HospitalUnited States

**Keywords:** dystonia, cholinergic, neurodegeneration, DYT1, torsinA, motor behavior, Mouse

## Abstract

Cholinergic dysfunction is strongly implicated in dystonia pathophysiology. Previously (Pappas et al., 2015;4:e08352), we reported that Dlx5/6-Cre mediated forebrain deletion of the DYT1 dystonia protein torsinA (Dlx-CKO) causes abnormal twisting and selective degeneration of dorsal striatal cholinergic interneurons (ChI) (Pappas et al., 2015). A central question raised by that work is whether the ChI loss is cell autonomous or requires torsinA loss from neurons synaptically connected to ChIs. Here, we addressed this question by using ChAT-Cre mice to conditionally delete torsinA from cholinergic neurons (‘ChAT-CKO’). ChAT-CKO mice phenocopy the Dlx-CKO phenotype of selective dorsal striatal ChI loss and identify an essential requirement for torsinA in brainstem and spinal cholinergic neurons. ChAT-CKO mice are tremulous, weak, and exhibit trunk twisting and postural abnormalities. These findings are the first to demonstrate a cell autonomous requirement for torsinA in specific populations of cholinergic neurons, strengthening the connection between torsinA, cholinergic dysfunction and dystonia pathophysiology.

## Introduction

Multiple lines of evidence implicate striatal cholinergic dysfunction in dystonia pathophysiology (Pappas et al., 2015; Albin et al., 2003; Eskow Jaunarajs et al., 2015; Pappas et al., 2014). The symptoms of DYT1 dystonia, caused by a loss of function mutation in the gene encoding torsinA (Ozelius et al., 1997), are reduced by antimuscarinic treatments (*e.g.,* trihexyphenidyl)(Burke et al., 1986). Antimuscarinic agents also reduce motor (Pappas et al., 2015) and electrophysiological (Maltese et al., 2014) abnormalities in DYT1 mouse models. Striatal cholinergic dysfunction is a common feature of multiple DYT1 animal models (Pappas et al., 2015; Martella et al., 2009; Pisani et al., 2006; Sciamanna et al., 2012a; Sciamanna et al., 2012b), and experimental ablation of striatal cholinergic interneurons (ChI) can lead to abnormal postures (Kaneko et al., 2000).

We demonstrated previously that deletion of torsinA from forebrain GABAergic and cholinergic neurons (using Dlx5/6-cre; ‘Dlx-CKO’) causes highly selective degeneration of dorsal striatal ChI roughly coincident with the juvenile onset of abnormal limb clasping and twisting movements(Pappas et al., 2015). Selective ChI abnormalities are also present in postmortem tissue from DYT1 subjects (Pappas et al., 2015). Abnormal movements in Dlx-CKO mice are reduced by clinically relevant antimuscarinic treatments, strengthening model therapeutic validity and suggesting shared pathophysiology with human dystonia. This work highlights the importance of elucidating the mechanism of selective ChI loss. A critical first step toward this goal is to determine whether the ChI loss observed in Dlx-CKO mice results from a cell autonomous role of torsinA in these cells or, alternatively, whether loss of torsinA from synaptically connected cells is also required. The major aim of these studies was to address this fundamental question.

To determine whether torsinA-related ChI loss is cell autonomous, we generated and characterized cholinergic neuron selective conditional torsinA knockout mice (ChAT-CKO). We find that ChAT-CKO mice phenocopy the selective degeneration of dorsal striatal ChI observed in Dlx-CKO mice (basal forebrain neuron numbers are normal in both models). Assessment of non-forebrain cholinergic populations demonstrates that pedunculopontine and laterodorsal tegmental brainstem cholinergic neurons, and spinal motor neurons also require torsinA for survival or normal function. ChAT-CKO mice exhibit severe motor and postural abnormalities that are distinct from Dlx-CKO mice. These findings are the first to establish a cell autonomous requirement for torsinA in ChI, as well as identifying additional vulnerable cholinergic neuron populations. This *in vivo* study fundamentally advances and expands understanding of the requirement of torsinA for normal cholinergic system function, opening new directions for the study of mechanisms contributing to selective neuronal dysfunction in dystonia.

## Results and discussion

**Figure 1.**
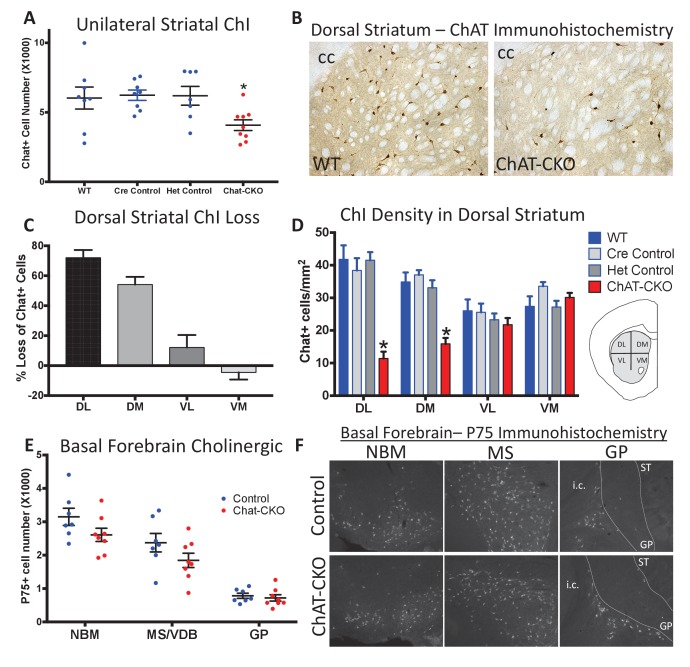
Conditional cholinergic neuron deletion of torsinA causes cell autonomous loss of striatal cholinergic neurons. (**A**) Unilateral stereological quantification of the number of ChAT-positive neurons in the striatum of ChAT-CKO and control mice (One-way ANOVA F_(3,28)_ = 3.589, p=0.02, Dunnett’s multiple comparisons test: adjusted p value = 0.049; ‘WT’=*Tor1a*^Flx/+^; ‘Cre Control’=ChAT-Cre+, *Tor1a* ^Flx/+^; ‘Het Control’=*Tor1 a*^Flx/-^; ‘ChAT-CKO’=ChAT-Cre+, *Tor1a*^Flx/-^). (**B**) ChAT immunohistochemistry of coronal sections containing dorsal striatum from WT and ChAT-CKO mice (cc = corpus callosum). (**C**) Percent reduction in cell density by striatal quadrant (DL = dorsolateral; DM = dorsomedial, VL = ventrolateral, VM = ventromedial). (**D**) Significant ChI loss is selective for dorsal striatal quadrants. Cell density quantification in control and ChAT-CKO striatal quadrants (Two-way ANOVA main effect of genotype F_(3,112)_ = 24.02, p<0.0001; main effect of quadrant F_(3,112)_=8.398, p<0.0001; interaction F_(9,112)_=8.11, p<0.0001. Post-hoc Tukey’s multiple comparisons test). (**E**) Basal forebrain neurons are spared in ChAT-CKO mice. Stereological quantification of P75-immunoreactive basal forebrain cholinergic neurons in the nucleus basalis of meynert (NBM), medial septum/nucleus of the vertical limb of the diagonal band (MS/VDB), and globus pallidus (GP). No differences in the number of cholinergic neurons was observed (NBM, t_(13)_=1.684, p=0.11; MS/VDB, t_(13)_=1.537, p=0.148; GP, t_(13)_=0.5, p=0.625). (**F**) P75 immunohistochemistry of sagittal sections containing basal forebrain cholinergic neuron populations. i.c. = internal capsule, ST = striatum.

To determine if ChI neurodegeneration is a cell autonomous effect of torsinA loss, we conditionally deleted torsinA from cholinergic neurons (*Chat-IRES-Cre^+^, Tor1a^Flx/-^*; ‘ChAT-CKO’ mice; Cre-recombinase expression occurs before birth and is completely selective for cholinergic neurons; Figure 1—figure supplement 1 [Madisen et al., 2010]). Unilateral unbiased stereology of ChAT-immunoreactive neurons in the dorsal striatum from 1 year old mice demonstrates a ~ 34% reduction in the number of dorsal striatal ChI in ChAT-CKO mice compared to control mice (Figure 1A,B). This finding was confirmed in an independent cohort using bilateral unbiased stereology (48% reduction; Figure 1—figure supplement 2A). The number of striatal non-cholinergic neurons was not different from controls (Figure 1—figure supplement 2B,C), demonstrating that there are no secondary cell loss effects of ChI degeneration, and that torsinA loss of function-mediated neurodegeneration is highly specific. These findings establish a cell autonomous torsinA requirement for ChI survival.

**Figure 1—figure supplement 1.**
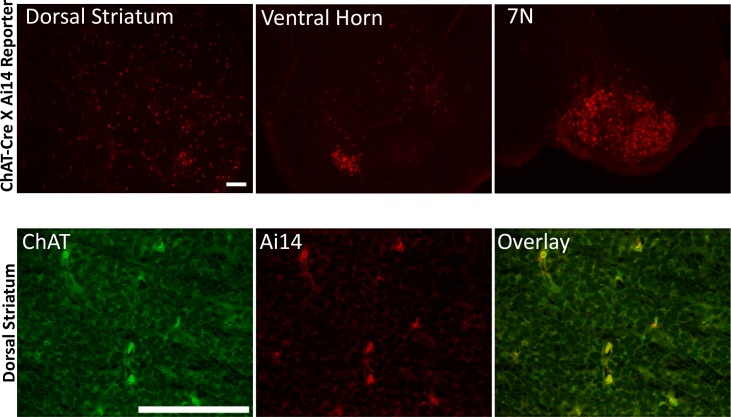
ChAT-Cre is expressed prenatally. (Upper panels) ChAT-Cre mice were crossed with Ai14 Cre reporter mice. Offspring were collected immediately after birth, brain sections were generated and observed under epifluorescence microscopy. (Lower panels) adjacent sections costained for torsinA. Scale bar = 50 μm.

**Figure 1—figure supplement 2.**
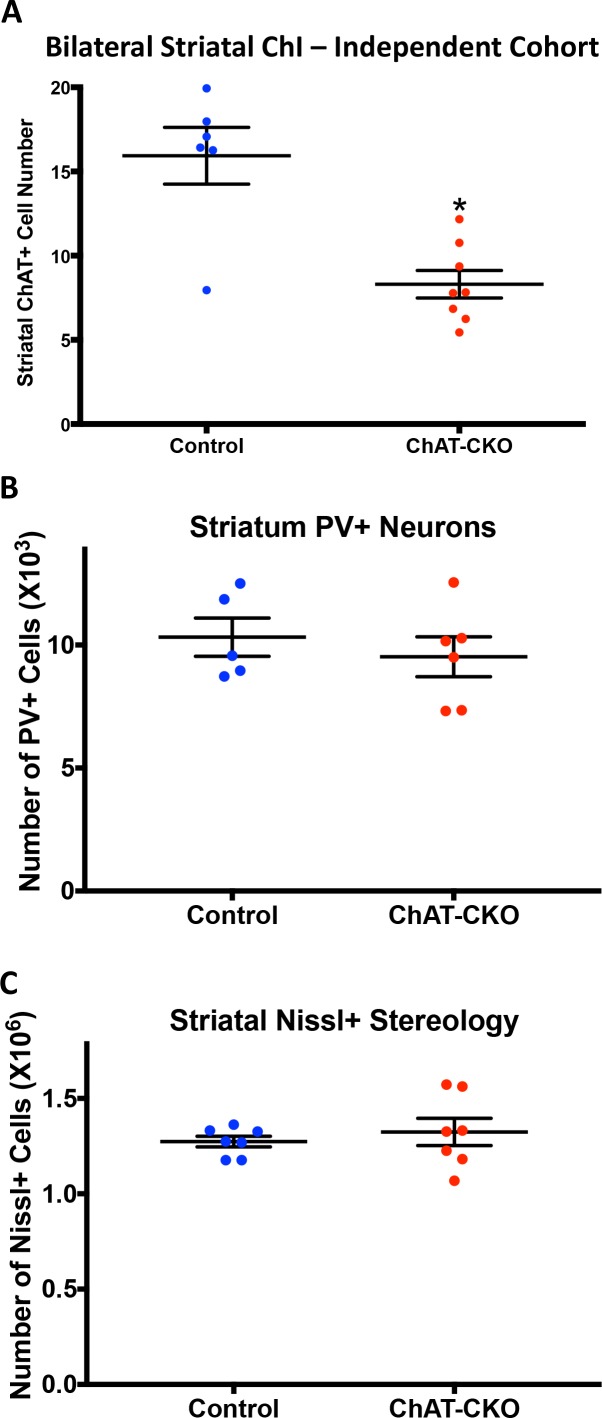
Independent cohort confirmation of selective striatal cholinergic neuron loss in ChAT-CKO mice. (**A**) Bilateral unbiased stereology of ChAT-immunoreactive neurons in the dorsal striatum (t_(12)_=4.42, p=0.0008). (**B**) Unbiased stereology of parvalbumin (PV) immunoreactive neurons in the dorsal striatum (t_9_ = 0.699, p=0.50). (**C**) Unbiased stereology of Nissl positive cells in the dorsal striatum (Welch’s t-test; t_7.803_=0.655, p=0.53).

**Figure 1—figure supplement 3.**
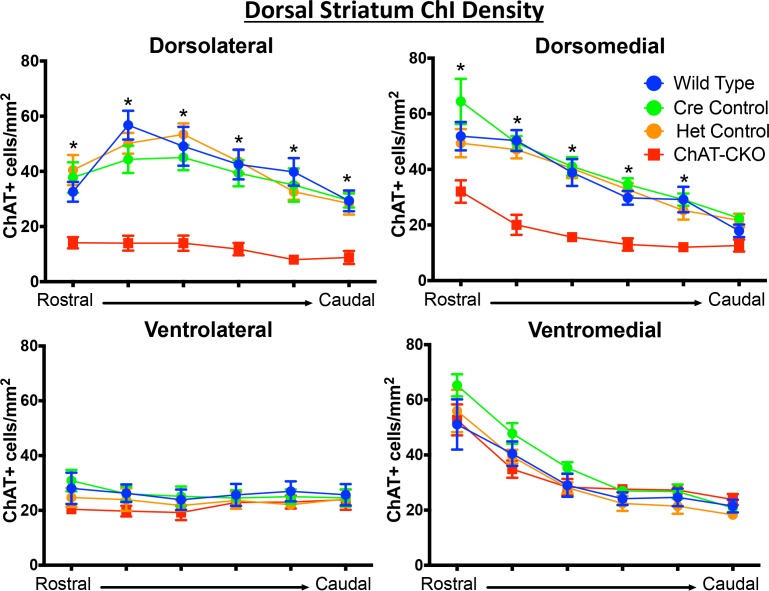
ChAT-positive neurons are reduced in a topographic pattern throughout the rostrocaudal extent of the dorsal striatum. Significant decreases in ChAT-positive cells were observed in the dorsolateral and dorsomedial segments of the dorsal striatum (dorsolateral striatum, two-way ANOVA main effect of genotype F_(3,156)_=74.77, p<0.0001, main effect of rostrocaudal section, F_(5,156)_=10.07, p<0.0001, no interaction F_(15,156)_=1.204, p=0.273; Dorsomedial striatum main effect of genotype F_(3,156)_=50.01, p<0.0001, main effect of rostrocaudal section, F_(5,156)_=41.81, p<0.0001, no interaction F_(15,156)_=1.646, p=0.067. Post-hoc Tukey’s test was performed for all significant main effects. * represents significant difference between ChAT-CKO mice and all control groups). No significant reductions were observed in the ventral segments of the dorsal striatum (ventrolateral; genotype F_(3,156)_=2.84, p=0.039 [post-hoc Tukey’s test adjusted p=0.129 or higher for all comparisons], rostrocaudal section F_(5,156)_=0.479, p=0.79, interaction F_(15,156)_=0.249, p=0.99. ventromedial; genotype F_(3,156)_= 2.706, p=0.047 [post-hoc Tukey’s test adjusted p=0.107 or higher for all comparisons], rostrocaudal section F_(5,156)_=46.28 p<0.0001, interaction F_(15,156)_=0.672, p=0.80).

**Figure 1—figure supplement 4.**
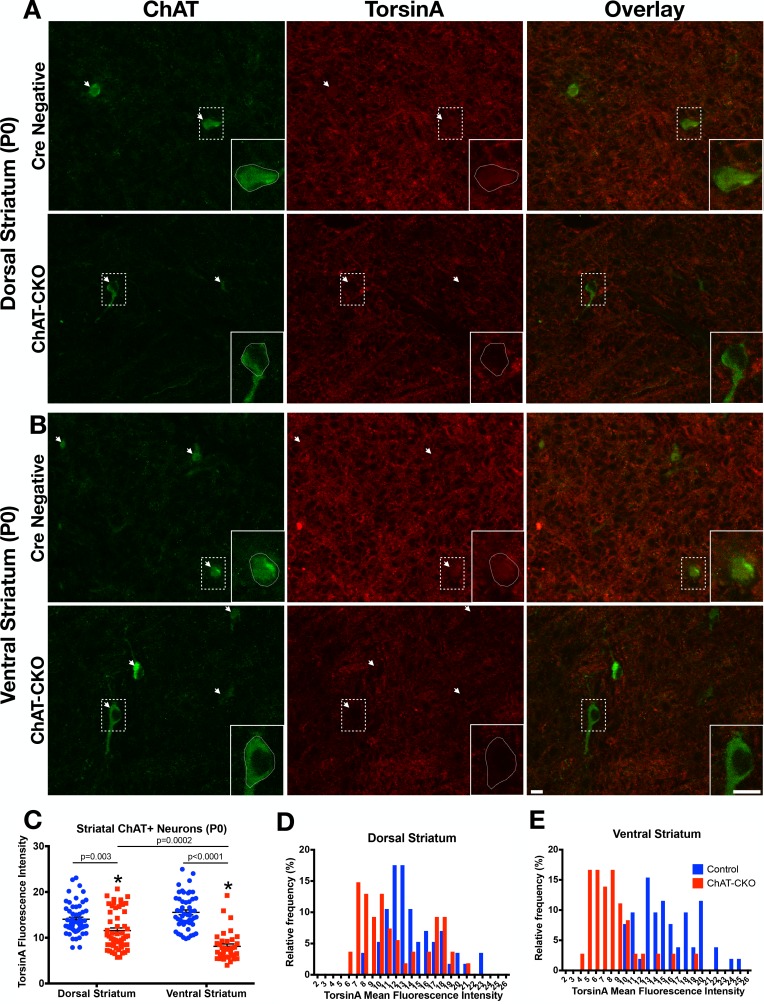
Time course of torsinA protein loss in dorsal and ventral striatum. (**A,B**) TorsinA and ChAT staining in dorsal and ventral striatum brain sections from P0 ChAT-CKO and control mice. (**C**) TorsinA mean fluorescence intensity analysis in dorsal or ventral striatal ChI (Two-way ANOVA main effect of genotype F_1,195_= 85.67, p<0.0001; Region, n.s., F_1,195_=3.301, p=0.07; interaction F_1,195_=21.34, p<0.0001. Sidak’s multiple comparisons test, Dorsal striatum control vs ChAT-CKO, p=0.003, Ventral striatum control vs ChAT-CKO, p<0.0001, ChAT-CKO dorsal striatum vs ventral striatum, p=0.0002; Control dorsal striatum vs ventral striatum, n.s., p=0.199). (**D,E**) Frequency histograms of torsinA mean fluorescence intensity in dorsal (n = 57 control, n = 54 ChAT-CKO neurons) and ventral striatal ChI (n = 52 control, n = 36 ChAT-CKO neurons). Scale bar = 10 µm.

**Figure 1—figure supplement 5.**
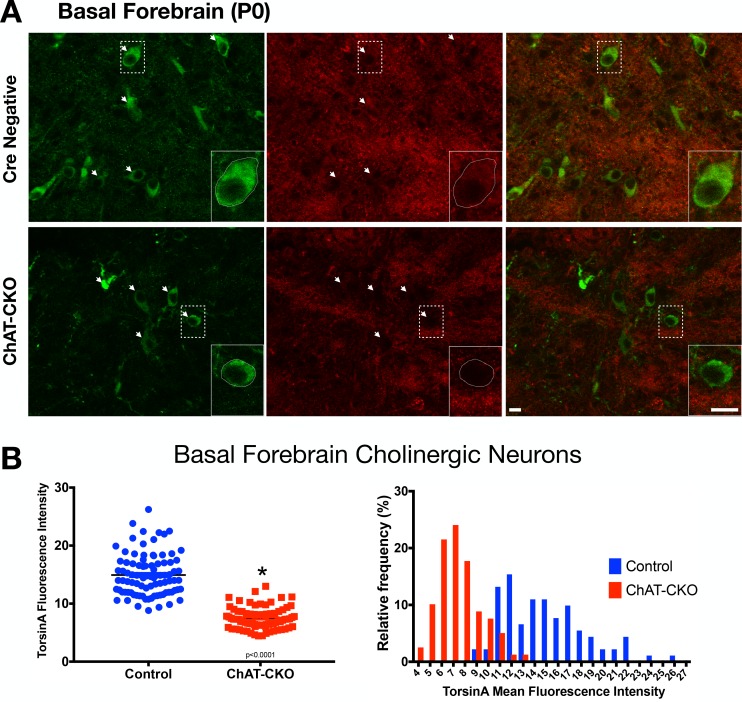
Time course of torsinA protein loss in basal forebrain. (**A**) TorsinA and ChAT staining in basal forebrain brain sections from P0 ChAT-CKO and control mice. (**B**) TorsinA mean fluorescence intensity analysis of control and ChAT-CKO (Welch’s t-test t_138.2_=17.35, p<0.0001) and frequency histograms of torsinA mean fluorescence intensity in basal forebrain cholinergic neurons ChI (n = 91 control, n = 79 ChAT-CKO neurons). Scale bar = 10 µm.

ChI cell loss is strikingly selective in Dlx-CKO mice, occurring primarily in the dorsal aspects of the striatum, with approximately six times greater cell loss in the dorsolateral compared to ventromedial striatum (57% vs 9% cell density reduction in Dlx-CKO mice; [Pappas et al., 2015]). To examine if the cell autonomous ChI degeneration in ChAT-CKO mice follows a similar subregion-selective pattern, we determined the density of ChAT-immunoreactive neurons in each quadrant of the dorsal striatum (as previously [Pappas et al., 2015]). Significant reductions in ChI number were limited to the dorsolateral and dorsomedial segments of the dorsal striatum (72% and 54% cell density reductions in dorsolateral and dorsomedial, vs 12% and −4% in ventrolateral and ventromedial segments; Figure 1C). This topographic pattern of cell loss was present throughout the entire rostro-caudal extent of the striatum (Figure 1C,D, Figure 1—figure supplement 3). The dorsolateral selectivity of ChI neuron loss is highly relevant, as the dorsolateral striatum is a key motor circuit node functionally integrated according to topographic inputs, whereas ventromedial striatal neurons are connected in associative and limbic circuits (Alexander et al., 1986; Haber, 2016; Parent and Hazrati, 1995). In contrast, the basal forebrain contains cholinergic projection neurons subserving cognitive and attentional control (Hasselmo and Sarter, 2011; Ballinger et al., 2016), which do not degenerate in either model (Figure 1E,F). Conditional deletion of torsinA from forebrain cholinergic neurons therefore mimics the region-selective vulnerability observed in Dlx-CKO mice, demonstrating a cell autonomous requirement for torsinA in select cholinergic populations. To determine if differing time courses of torsinA loss (via differing torsinA half lives) contributes to selective vulnerability, we assessed torsinA levels in dorsal vs ventral striatal ChI at P0. Surprisingly, despite uniform prenatal Cre recombinase expression and preferential loss of dorsal ChI, torsinA levels were reduced to a greater extent in ventral ChI (dorsal ChI contained 82% of control torsinA levels, while ventral ChI had ~52% remaining; Figure 1—figure supplement 4). Non-vulnerable basal forebrain cholinergic neurons exhibited 49% of control torsinA immunoreactivity (Figure 1—figure supplement 5). These findings demonstrate that a more rapid loss of torsinA during development does not contribute to the unique vulnerability of dorsal ChI.

**Figure 2.**
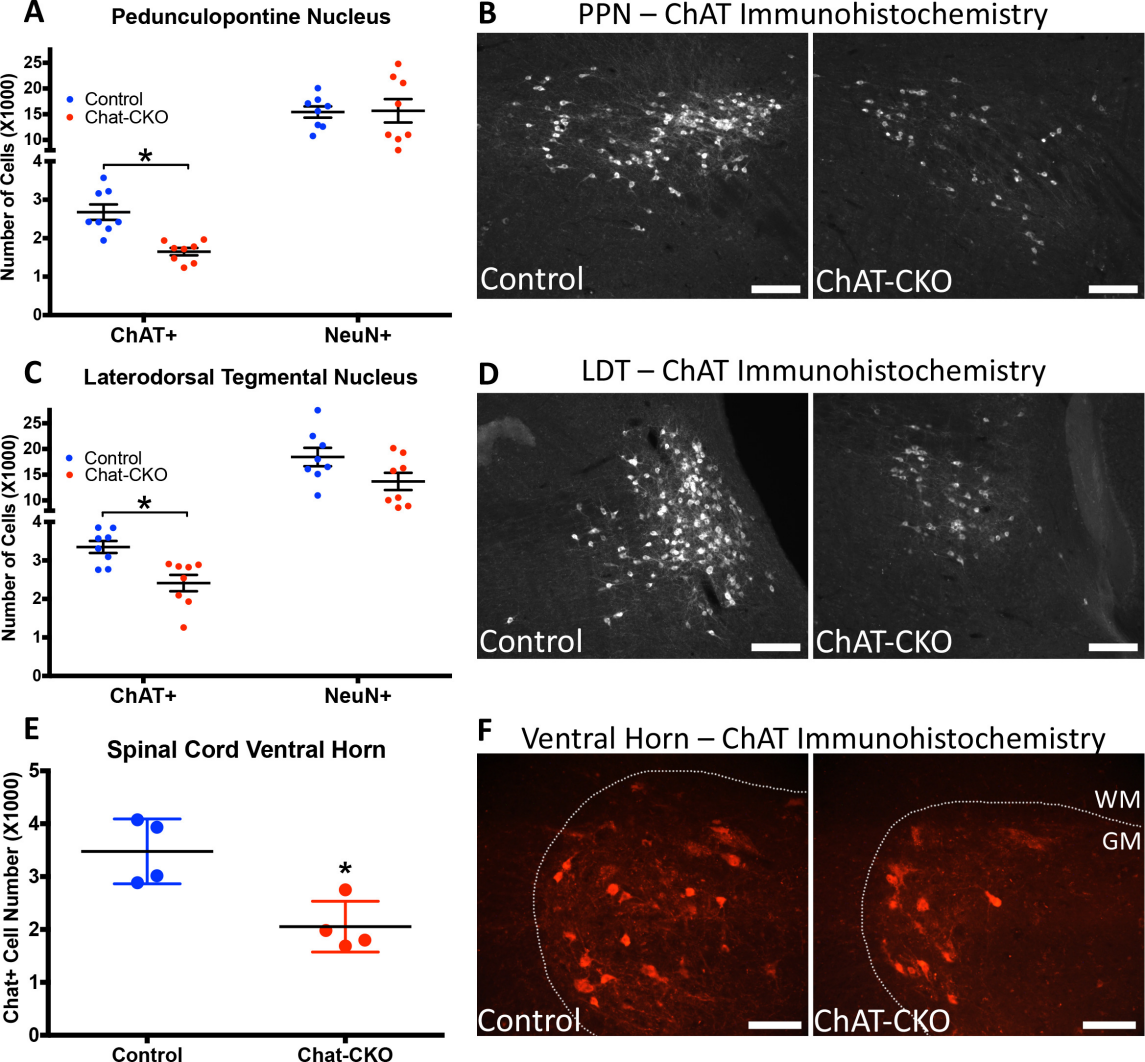
ChAT-CKO mice have significantly fewer brainstem and spinal cord cholinergic neurons. (**A,B**) Stereological quantification of ChAT-positive or NeuN-positive neurons in the pedunculopontine nucleus (PPN) of control and ChAT-CKO mice (ChAT; t_(14)_=4.531, p=0.0005. NeuN; t_(14)_=0.095, p=0.92). (**C,D**) Stereological quantification of ChAT-positive or NeuN-positive neurons in the laterdorsal tegmental nucleus (LDT) of control and ChAT-CKO mice (ChAT; t_(14)_=3.571, p=0.003. NeuN; t_(14)_=1.934, p=0.073). (**E,F**) Quantification of the number of ChAT-positive neurons in the cervical spinal cord of control and ChAT-CKO mice (t_(6)_=3.654, p=0.0107). Scale bars = 100 μm.

TorsinA deletion is restricted to forebrain structures in Dlx-CKO mice. In contrast, ChAT-CKO mice lack torsinA in all cholinergic neurons throughout the neuraxis, enabling us to assess the impact of torsinA loss in additional cholinergic populations. Unbiased stereology of ChAT-immunoreactive neurons in the brainstem demonstrates significantly fewer cholinergic neurons in the pedunculopontine (PPN) and laterodorsal tegmental (LDT) nuclei in 1 year old Chat-CKO mice (Figure 2A–D). The PPN and LDT also contain GABAergic, and glutamatergic neurons (Mena-Segovia, 2016), which significantly outnumber cholinergic neurons (Mena-Segovia et al., 2009; Wang and Morales, 2009). Unbiased stereology of NeuN +neurons in PPN and LDT showed no significant change in the overall number of neurons (Figure 2A,C). Because cholinergic neurons are a minority of cells in the PPN and LDT, it is possible that a significant reduction of this small sub-population cannot be detected when assessed by counting overall NeuN +neuron number. It is also possible that PPN and LDT cholinergic neurons exhibit reduced ChAT expression rather than actual cell loss. Regardless, either possibility demonstrates a cell autonomous role for torsinA for normal function of these cells. These findings also indicate that the loss or dysfunction of brainstem cholinergic neurons does not have deleterious effects on the viability of surrounding neurons. Consistent with this finding, there was no evidence of reactive microgliosis or astrogliosis in the brainstem (Figure 2—figure supplement 1). Quantification of the number of spinal motor neurons (C3-C5; [Kim et al., 2017]) demonstrated significantly fewer motor neurons in ChAT-CKO mice (Figure 2E,F).

**Figure 2—figure supplement 1.**
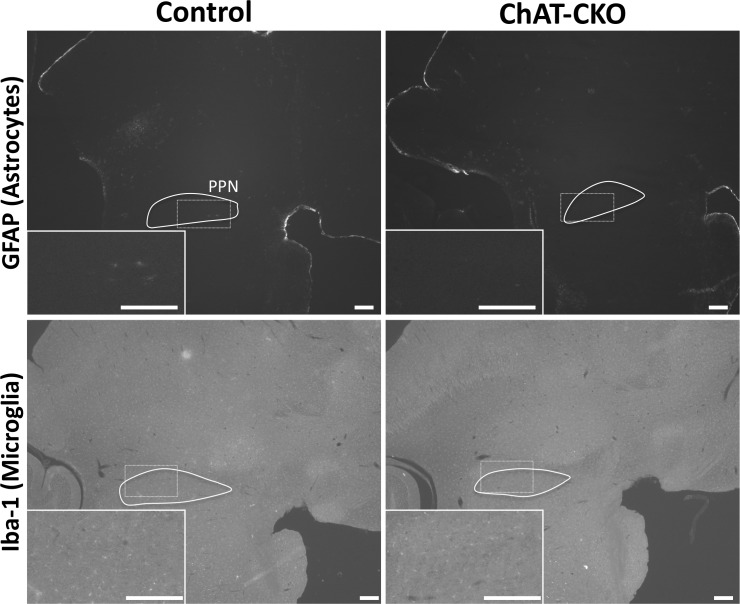
Absence of gliosis in the brainstem of ChAT-CKO mice. Immunohistochemistry of GFAP (glial fibrillary acidic protein; specific to astrocytes) and Iba-1 (ionized calcium-binding adapter molecule 1; specific to microglia) in sagittal sections of control and ChAT-CKO hindbrain demonstrate normal distribution of glia.

The identification of cholinergic dysfunction or loss in PPN and LDT is notable, as considerable data implicate these cells in motor and postural control. PPN and LDT cholinergic neurons are distributed in a rostrocaudal continuum in the brainstem, forming a coordinated functional unit (Mena-Segovia, 2016; Mena-Segovia and Bolam, 2017). PPN and LDT cholinergic neurons topographically innervate the striatum and striatal-projecting thalamic and midbrain dopamine neurons (Dautan et al., 2014), such that rostral PPN modulates motor-related circuits, LDT innervates limbic circuits, and caudal PPN targets both regions (Mena-Segovia, 2016; Xiao et al., 2016) via both direct and indirect inputs. Consistent with a central role in modulating locomotor activity, optogenetic stimulation of PPN cholinergic neurons alters locomotion speed, while stimulation of adjacent glutamatergic neurons induces locomotion (Xiao et al., 2016; Roseberry et al., 2016; Capelli et al., 2017). Cholinergic PPN lesion alone or in combination with dopaminergic denervation impairs gait and causes postural abnormalities in primates (Grabli et al., 2013; Karachi et al., 2010). In rodents, cholinergic-selective PPN lesion impairs performance on the accelerating rotarod and alters sensorimotor gating (MacLaren et al., 2014a; MacLaren et al., 2014b), while non-specific PPN ablation alters gait (Blanco-Lezcano et al., 2017) and impairs reversal learning (Syed et al., 2016). Human neuroimaging and postmortem studies also provide support for a connection between PPN cholinergic integrity and motor function. PPN cholinergic loss is linked to gait abnormalities in Parkinson disease (Karachi et al., 2010; Bohnen et al., 2009), and brainstem lesions (including PPN loss) can result in complex dystonia (Jankovic and Patel, 1983; LeDoux and Brady, 2003; Loher and Krauss, 2009; Zweig et al., 1988; Mente et al., 2018). Systematic cholinergic brainstem cell counts have not been performed in DYT1 dystonia postmortem samples; most studies have failed to demonstrate neuronal inclusions or overt cell loss in this region (Paudel et al., 2014; Pratt et al., 2016; McNaught et al., 2004).

**Figure 3.**
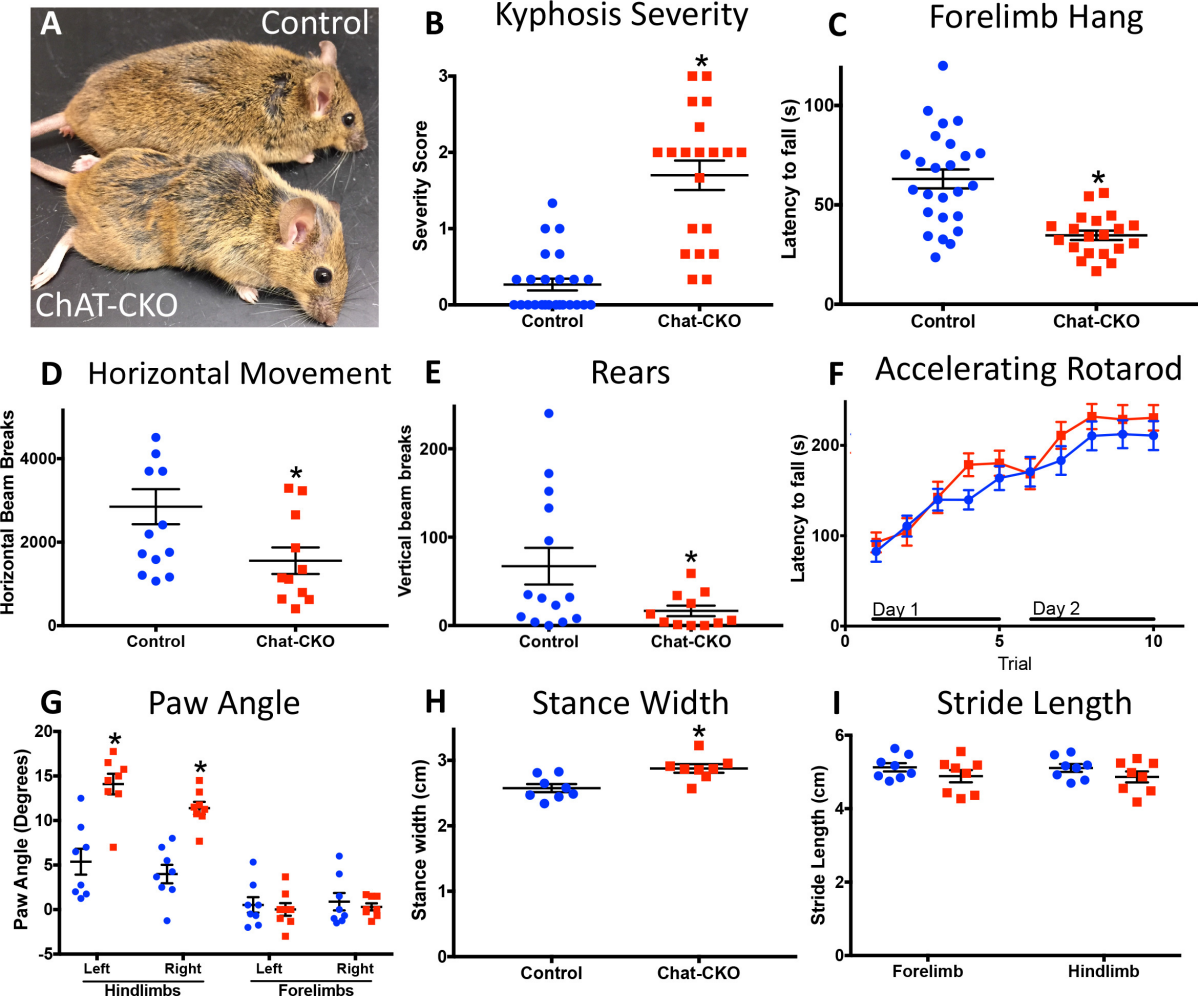
Motor behavior is severely disrupted in ChAT-CKO mice. (**A**) Representative image of a control and ChAT-CKO mouse demonstrates severe kyphosis and unkempt coat. (**B**) ChAT-CKO mice exhibit significantly increased kyphotic curvature during locomotion (Mann-Whitney U = 35, p<0.0001). (**C**) ChAT-CKO mice exhibit a significantly reduced latency to fall during forelimb suspension (Mann-Whitney U = 71.5, p<0.0001). (**D, E**) ChAT-CKO mice are hypoactive in the open field (horizontal movement, t_(23)_=2.345, p=0.028; vertical rears, welch-corrected t_(15.1)_ = 2.345, p=0.033). (**F**) Performance on the accelerated rotarod does not significantly differ from controls (two-way repeated measures ANOVA, genotype, F_(1,43)_=0.75, p=0.389; trial, F_(9,387)_=55.63, p<0.0001; interaction, F_(9,387)_=1.194, p=0.297). (G - I) ChAT-CKO mouse gait is abnormal during locomotion (*paw angle*, two-way ANOVA main effect of genotype, F_(1,56)_=30.54, p<0.0001, main effect of limb F_(3,56)_=51.02, p<0.0001, interaction F_(3,56)_=13.51, p<0.0001, post-hoc Sidak’s multiple comparisons test. *Stance width*, t_(14)_=3.329, p=0.005. *Stride length*, two-way ANOVA genotype F_(1,28)_=3.164, p=0.086, limb F_(1,28)_=0.02, p=0.887, interaction F_(1,28)_=0.0001, p=0.989).

Motor behavior is severely disrupted in ChAT-CKO mice, but is distinct from the Dlx-CKO phenotype (Figure 3; Table 1). ChAT-CKO pups are initially indistinguishable from littermates, but at approximately 4 weeks of age develop a hunched posture, have unkempt fur, and exhibit reduced responsiveness to handling (Figure 3A, Figure 3—figure supplement 1). Whereas normal mice exhibit a slight dorsal spinal curvature at rest, ChAT-CKO mice exhibit severe kyphosis, including during locomotion (assessed by two observers blind to experimental conditions; Figure 3B; Figure 3—figure supplement 1; Figure 3—video 1) (Guyenet et al., 2010). ChAT-CKO mice also exhibit signs of weakness, including a significantly reduced ability to hang by the forelimbs (Figure 3C), tremulous movements, labored breathing (Figure 3—video 1), and significantly reduced horizontal and vertical movement in the open field (Figure 3D,E, Figure 3—figure supplement 2). Remarkably, performance on the accelerating rotarod during two days of training appears normal (Figure 3F). The normal rotarod behavior differs from models of motor neuron and neuromuscular disease, suggesting that neuromuscular weakness is modest in ChAT-CKO, and less likely to contribute to other behavioral phenotypes (e.g., postural abnormality). The gait of ChAT-CKO mice is also significantly altered (Figure 3G–I). This constellation of behavioral phenotypes is distinct from Dlx-CKO mice (Table 1), in which loss of dorsal striatal ChI is associated with a set of persistent abnormal action-induced motor behaviors, including limb clasping and trunk twisting during tail suspension and open field hyperactivity (Pappas et al., 2015). ChAT-CKO mice did not exhibit fore- or hindlimb clasping during tail suspension, but did exhibit tremulousness and trunk twisting (15 CKO, 19 heterozygous, 22 Cre control, and 19 wild type mice observed; Figure 3—video 2). These results suggest that dorsal striatal ChI neurodegeneration may not, by itself, be sufficient to cause limb clasping during tail suspension. However, the co-occurrence of brainstem and spinal cord neurodegeneration and tremulousness in ChAT-CKO mice could modify a clasping phenotype and therefore limit this strength of this conclusion.

**Figure 3—figure supplement 1.**
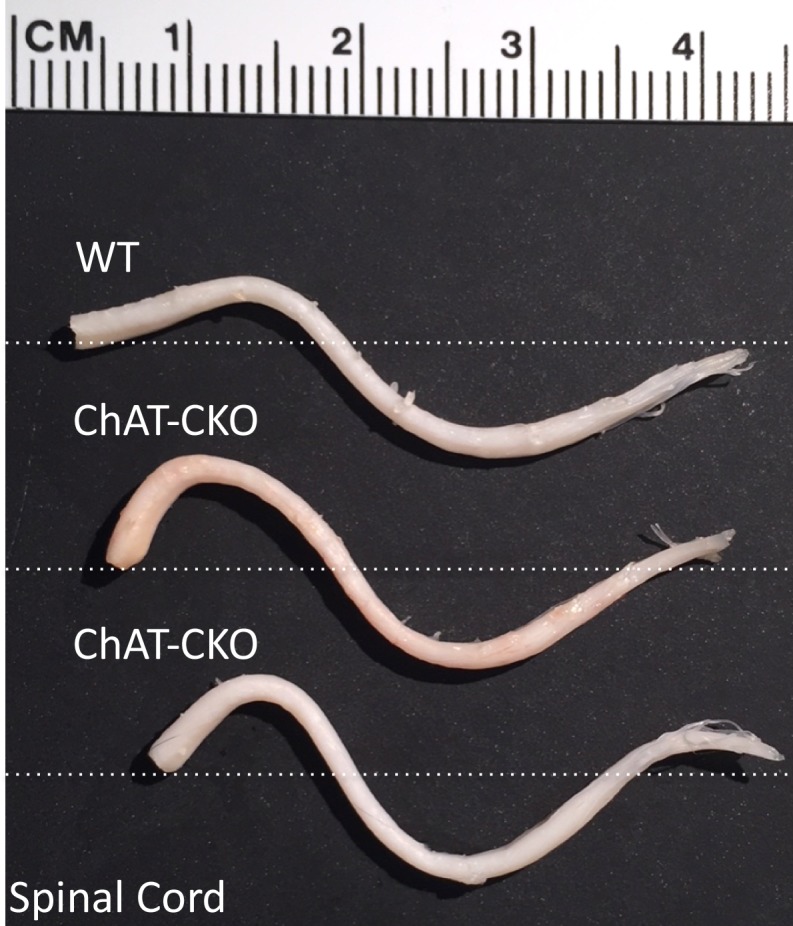
Representative examples of control and ChAT-CKO spinal cords demonstrate significant kyphotic curvature.

**Figure 3—figure supplement 2.**
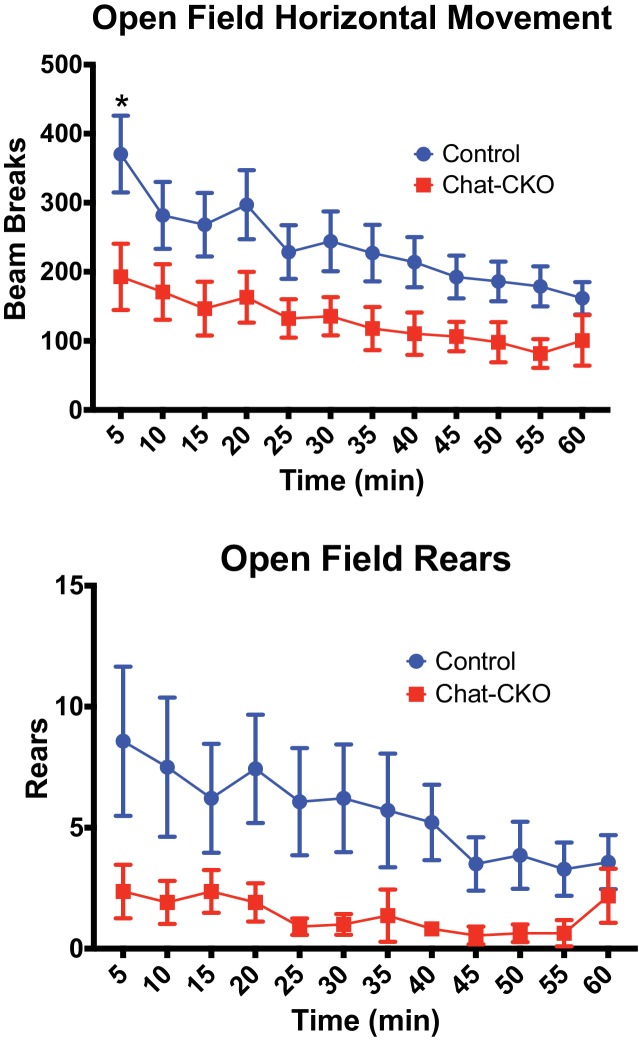
ChAT-CKO mice are significantly hypoactive. (Upper panel) Open field analysis of horizontal movements (two-way repeated measures ANOVA main effect of genotype F_(1,23)_=5.498, p=0.02, time F_(11,253)_=9.222, p<0.0001, interaction F_(11,253)_=0.899, p=0.541, post-hoc Sidak’s multiple comparisons test). (Lower panel) Open field analysis of vertical rearing movements (two-way repeated measures ANOVA main effect of genotype F_(1,23)_=4.413, p=0.046, time F_(11,253)_=2.452, p=0.0063, interaction F_(11,253)_=0.987, p=0.458).

**Figure 3—video 1.** Representative video demonstrating tremulousness, kyphosis, and hyperactivity in ChAT-CKO mice, as compared to controls.

**Figure 3—video 2.** ChAT-CKO exhibit twisting and tremulousness, but not limb clasping during tail suspension. Representative video demonstrating tail suspension test in control and ChAT-CKO mice.

**Table 1.** Behavioral properties of Dlx-CKO and ChAT-CKO mice.

Motor function	Dlx-CKO	ChAT-CKO
	Pappas et al., 2015 eLife 4:e08352	present manuscript
Tail suspension	Trunk twisting	Trunk twisting
	Forelimb clasping	-
	Hindlimb clasping	-
	-	Tremulousness
Open field	Hyperactivity	Hypoactivity
Rotarod	Normal	Normal
Response to handling	Exaggerated	Reduced
Weakness, latency to fall	Grid hang reduction	Wire hang reduction
Gait	Normal by eye	Abnormal by eye
	Slightly reduced stance width	Increased stance width
	-	Increased paw angle
Overt postural abnormalities	-	Severe kyphosis
Tremulous movement	-	Severe
Labored breathing	-	Severe

While no single system or experimental approach can fully model a disease, the extreme postural abnormalities (kyphosis and twisting) in ChAT-CKO mice are reminiscent of Oppenheim’s original description of dystonia (Klein and Fahn, 2013), suggesting that a constellation of cholinergic abnormalities may contribute to such a phenotype. The abnormal gait, tremulous movement, weakness, labored breathing, and appearance of reduced muscle mass in ChAT-CKO mice are consistent with brainstem and spinal cord pathology, yet the time course of ChAT-CKO abnormalities (beginning during development) differ from motor neuron and neuromuscular disease models, in which behavioral phenotypes typically emerge in adulthood (9–11 months of age; (Dickinson and Meikle, 1973; Bridges et al., 1992; Deconinck et al., 1997; Grady et al., 1997; Laws and Hoey, 2004; Liu et al., 2016; Sopher et al., 2004; Monks et al., 2007). Early motor behavioral manifestations also occur in Dlx-CKO and other DYT1 models, emphasizing the importance of torsinA function during development and maturation at behavioral (Pappas et al., 2015; Liang et al., 2014), cellular (Pappas et al., 2018), and molecular levels (Tanabe et al., 2016).

These findings establish a cell autonomous requirement of torsinA for the normal function and survival of distinct populations of cholinergic neurons. Comparison of basic cellular properties between susceptible and invulnerable cholinergic neuron populations does not identify obvious patterns driving selective vulnerability (Tables 2 and 3). Within the striatum, dorsal ChI are highly vulnerable to cell death, while ventral ChI are spared. It is unclear whether molecular differences within different ChI populations drive vulnerability, or if differences in connectivity or response to inputs contributes to their loss; these possibilities are not mutually exclusive. While often considered a single neuronal class, an existing and enlarging literature demonstrates that dorsal and ventral striatal ChI exhibit significant differences in morphology, regulation, and receptor expression (reviewed in [Gonzales and Smith, 2015]), as well as differing firing patterns during behavioral tasks (Yarom and Cohen, 2011) and responses to serotonergic input (Virk et al., 2016). These differences implicate the presence of multiple ChI subclasses, though it is important to note that the spared ‘ventral’ population here represents the ventral part of the dorsal striatum, not the nucleus accumbens. Thalamostriatal and corticostriatal input is highly topographic (Alexander et al., 1986; Smith et al., 2004), raising the possibility that aberrant input from different thalamic nuclei or cortical regions (or aberrant response to that input) could alter the susceptibility of dorsal vs ventral ChI. It is likely that a combination of these and other factors plays a role in the differential susceptibility of cholinergic neuronal populations, including their molecular profiles (e.g., protective factors in some neurons, susceptibility factors in others), the response to afferent inputs, and their inherent physiological properties.

**Table 2.** Vulnerability of cholinergic populations. (*)=Unconfirmed by independent marker.

	Cre expression		Cell death vulnerability	
Cholinergic population	Dlx-Cre	ChAT-Cre	Dlx-Cre	ChAT-Cre
Dorsolateral striatum (including dorsal caudate putamen)	Confirmed	Confirmed	Severe	Severe
Dorsomedial striatum (including ventral caudate putamen)	Confirmed	Confirmed	Mild	Spared
Nucleus accumbens	Confirmed	Confirmed	-	-
Basal forebrain	Confirmed	Confirmed	Spared	Spared
Cholinergic Brainstem	Absent	Confirmed	n/a	Severe (*)
Primary Motor Neurons	Absent	Confirmed	n/a	Moderate

**Table 3.** Properties of cholinergic neuronal populations. ‘Nucleus Basalis Complex’=Nucleus Basalis of Meynert, Horizontal limb of the diagonal band of Broca, Ventral Pallidum, Magnocellular Preoptic Area, Substantia Inominata, Nucleus of the Ansa Lenticularis. ‘Septa”l = Medial Septum, Vertical Limb of the Diagonal Band of Broca. ‘Cholinergic Brainstem’=Pedunculopontine Nucleus, Laterodorsal Tegmental Nucleus (Pappas et al., 2015; Mena-Segovia and Bolam, 2017; Gonzales and Smith, 2015; Manns et al., 2000; Unal et al., 2012; Petzold et al., 2015; Kanning et al., 2010; Kreitzer, 2009; Zaborszky et al., 2012; Garcia-Rill, 1991; Semba et al., 1988; Semba and Fibiger, 1992; Phelps et al., 1990a; Phelps et al., 1988; Phelps et al., 1990b; Phelps et al., 1989; Aroca and Puelles, 2005; Schambra et al., 1989).

Cholinergic population	Neuronal class	Firing properties	Efferent projections	Afferent inputs	Birth date/final mitosis	Embryonic origin	ChAT expression
Dorsolateral striatum (including dorsal caudate putamen)	Interneuron	tonically active, 2–10 Hz baseline firing rate	Local - striatal spiny projection neurons and fast spiking interneurons	Thalamus, sensorimotor cortex, striatal spiny projection neurons, striatal interneurons	E12-E15	MGE	~E16
Dorsomedial striatum (including ventral caudate putamen)	Interneuron	tonically active, 2–10 Hz baseline firing rate	Local - striatal spiny projection neurons and fast spiking interneurons	Thalamus, association cortices, striatal spiny projection neurons, striatal interneurons	E12-E15	MGE	~E16
Nucleus accumbens	Interneuron	tonically active, 0.6–12 Hz baseline firing rate	Local - striatal spiny projection neurons and fast spiking interneurons	Thalamus, frontal cortex, striatal spiny projection neurons, striatal interneurons	E12-E15	MGE	~E16
Basal forebrain	Projection neuron	Tonic/burst, subtype dependent	Cortex (Nucleus Basalis Complex), Hippocampus (Septal)	Medulla, locus ceruleus, substantia nigra, ventral tegmental area, hypothalamic nuclei, nucleus accumbens, amygdala, local intrinsic GABAergic and glutamatergic collaterals	E11-E15	POA/MGE	~E15-16
Cholinergic Brainstem	Projection neuron	episodic	Midbrain, superior colliculus, thalamus, globus pallidus, hypothalamus, septum, striatum, cortex	Brainstem reticular formation, midbrain central gray, lateral hypothalamus-zona incerta, cortex, amygdala, basal forebrain, basal ganglia output nuclei, brainstem and spinal cord sensory nuclei	E12-E13	Ventral rhombomere 1 (r1)	
Primary Motor Neurons	Projection neuron	subtype dependent	Muscle	Motor Cortex, local spinal cord interneurons and sensory neurons	E11-E12	Ventral spinal cord progenitor domains	E13

These studies greatly strengthen the connection between torsinA and cholinergic dysfunction, demonstrating that specific cholinergic populations exhibit a cell autonomous selective vulnerability to torsinA deficiency, while others – basal forebrain and ventral striatum – are spared. These findings open novel avenues of study aimed at defining the molecular mechanisms responsible for this cell autonomous selective vulnerability, and circuit-level analyses to ameliorate the effects of cholinergic neurotransmission abnormalities.

## Materials and methods

**Key resources table:** 

Reagent type (species) or resource	Designation	Source or reference	Identifiers	Additional information
Gene (Mus musculus)	*Tor1a*	NA	NCBI Gene: 30931; MGI:1353568	Encodes TorsinA
Strain, strain background(M. musculus)	ChAT-Cre	Jackson Laboratories	Stock ID 006410	Chat^tm2(cre)Lowl^; (Chat-IRES-Cre)
Strain, strain background(M. musculus)	Tor1a^Flx/Flx^	Jackson Laboratories	Stock ID 025832	Tor1a^tm3.1Wtd^
Strain, strain background(M. musculus)	Tor1a^-/-^	Jackson Laboratories	Stock ID 006251	Tor1a^tm1Wtd^
Antibody	Choline Acetyltransferase	Millipore AB144P	RRID: AB_2079751	1:100
Antibody	P75 Neurotrophin Receptor	Santa Cruz sc6188	RRID: AB_2267254	1:100
Antibody	NeuN	Cell Signaling #12943	RRID: AB_2630395	1:500
Antibody	GFAP	Cell Signaling #3670P	RRID: AB_561049	1:1000
Antibody	Iba-1	Wako 019–19741	RRID: AB_839504	1:500
Antibody	TorsinA	Abcam ab34540	RRID: AB_2240792	1:100
Antibody	anti-mouse	ThermoFisher A-31571	RRID: AB_162542	1:800
Antibody	anti-rabbit	ThermoFisher A-21206	RRID: AB_2535792	1:800
Antibody	anti-rabbit	ThermoFisher A-31572	RRID: AB_162543	1:800
Antibody	anti-goat	ThermoFisher A-21432	RRID: AB_2535853	1:800
Antibody	anti-goat	Jackson Immunoresearch 705-065-003	RRID: AB_2340396	1:800
Commercial assay or kit	ABC HRP Kit (Standard)	Vector Laboratories	Pk-6100	Vectastain elite ABC kit

### Animals

ChAT-CKO mice were generated by crossing *Chat^tm2(cre)Lowl^* mice (Rossi et al., 2011) with *Tor1a^Flx/Flx^* mice (Liang et al., 2014), using the breeding strategy described in (Pappas et al., 2015), and maintained as previously described (Pappas et al., 2015).

### Sample size estimation

Sample sizes for histological and behavioral studies were determined by performing a power analysis of the open field or striatal cholinergic stereological data (mean and std. dev.) from (Pappas et al., 2015), an alpha of 0.01, and beta of 0.1. (Kane SP. Sample Size Calculator. ClinCalc: *http://clincalc.com/stats/samplesize.aspx*). Experimental cohorts were generated accordingly.

### Imaging and stereology

Brain sections were generated and stained with immunohistochemistry using the methods described in (Pappas et al., 2015; Pappas et al., 2018). Antibodies and reagents are listed in Table 4. Sections were observed with epifluorescence or brightfield microscopy (Pappas et al., 2018), and unbiased stereological cell counting was performed with StereoInvestigator software using the Optical Fractionator probe (specific parameters in Table 5). Striatal cell density was quantified as done previously (Pappas et al., 2015). Spinal cord neurons were quantified as described in (Kim et al., 2017).

**Table 4.** Antibodies used for immunohistochemistry.

Level	Antigen	Host	Conjugated	Dilution	Source
Primary	Choline Acetyltransferase	Goat	-	1:100	Millipore AB144P
Primary	P75 Neurotrophin Receptor	Goat	-	1:100	Santa Cruz sc6188
Primary	NeuN	Rabbit	-	1:500	Cell Signaling #12943
Primary	GFAP	Mouse	-	1:1000	Cell Signaling #3670P
Primary	Iba-1	Rabbit	-	1:500	Wako 019–19741
Primary	TorsinA	Rabbit	-	1:100	Abcam ab34540
Secondary	anti-mouse	Donkey	Alexafluor-647	1:800	ThermoFisher A-31571
Secondary	anti-rabbit	Donkey	Alexafluor-488	1:800	ThermoFisher A-21206
Secondary	anti-rabbit	Donkey	Alexafluor-555	1:800	ThermoFisher A-31572
Secondary	anti-goat	Donkey	Alexafluor-555	1:800	ThermoFisher A-21432
Secondary	anti-goat	Donkey	biotin	1:800	Jackson Immunoresearch 705-065-003

**Table 5.** Stereology parameters.

Region	Marker	Counting frame (μm)	Grid size (μm)	Guard zone (μm)	Dissector (μm)	Section cut thickness (μm)
Striatum	ChAT	100 × 100	250 × 250	1	10	40
NBM	P75	90 × 90	200 × 200	5	30	50
MS/VDB	P75	90 × 90	200 × 200	5	30	50
GP	P75	100 × 100	140 × 140	5	30	50
PPN and LDT	ChAT	75 × 75	150 × 150	5	30	50
PPN and LDT	NeuN	40 × 40	250 × 250	5	30	50

### Behavioral analysis

Tail suspension, forelimb wire suspension, open field, accelerating rotarod, and gait analysis were performed as described in (Pappas et al., 2015). Kyphosis severity was scored as described in (Guyenet et al., 2010).

### Statistical analysis

t-tests, one-way, or two-way ANOVA with posthoc corrections for multiple comparisons were performed to compare experimental groups (details in each figure legend). If variances were significantly different between groups, non-parametric tests were performed.

## Additional files

Transparent reporting form

### Data availability

All data generated during this study are included in the manuscript and supporting files
